# Docetaxel-Loaded Self-Assembly Stearic Acid-Modified *Bletilla striata* Polysaccharide Micelles and Their Anticancer Effect: Preparation, Characterization, Cellular Uptake and In Vitro Evaluation

**DOI:** 10.3390/molecules21121641

**Published:** 2016-12-02

**Authors:** Qingxiang Guan, Dandan Sun, Guangyuan Zhang, Cheng Sun, Miao Wang, Danyang Ji, Wei Yang

**Affiliations:** 1 Department of Pharmaceutics, School of Pharmacy, Jilin University, No. 1266, Fujin Road, Changchun 130021, China; guanqx@jlu.edu.cn (Q.G.); sundd15@mails.jlu.edu.cn (D.S.); zhangguangyuan11@126.com (G.Z.); suncheng15@mails.jlu.edu.cn (C.S.); wangmiao15@mails.jlu.edu.cn (M.W.); jidy16@mails.jlu.edu.cn (D.J.); 2 Department of Neurology, Second Hospital of Jilin University, Changchun 130041, China

**Keywords:** docetaxel, *Bletilla striata* polysaccharide, copolymer micelle, hemolysis assay, anticancer activity, apoptotic

## Abstract

Poorly soluble drugs have low bioavailability after oral administration, thereby hindering effective drug delivery. A novel drug-delivery system of docetaxel (DTX)-based stearic acid (SA)-modified *Bletilla striata* polysaccharides (BSPs) copolymers was successfully developed. Particle size, *zeta* potential, encapsulation efficiency (EE), and loading capacity (LC) were determined. The DTX release percentage in vitro was determined using high performance liquid chromatography (HPLC). The hemolysis and in vitro anticancer activity were studied. Cellular uptake and apoptotic rate were measured using flow cytometry assay. Particle size, *zeta* potential, EE and LC were 125.30 ± 1.89 nm, −26.92 ± 0.18 mV, 86.6% ± 0.17%, and 14.8% ± 0.13%, respectively. The anticancer activities of DTX-SA-BSPs copolymer micelles against HepG2, HeLa, SW480, and MCF-7 (83.7% ± 1.0%, 54.5% ± 4.2%, 48.5% ± 4.2%, and 59.8% ± 1.4%, respectively) were superior to that of docetaxel injection (39.2% ± 1.1%, 44.5% ± 5.3%, 38.5% ± 5.4%, and 49.8% ± 2.9%, respectively) at 0.5 μg/mL drug concentration. The DTX release percentage of DTX-SA-BSPs copolymer micelles and docetaxel injection were 66.93% ± 1.79% and 97.06% ± 1.56% in two days, respectively. Cellular uptake of DTX-FITC-SA-BSPs copolymer micelles in cells had a time-dependent relation. Apoptotic rate of DTX-SA-BSPs copolymer micelles and docetaxel injection were 73.48% and 69.64%, respectively. The SA-BSPs copolymer showed good hemocompatibility. Therefore, SA-BSPs copolymer can be used as a carrier for delivering hydrophobic drugs.

## 1. Introduction

Over the past decades, self-assembled copolymer micelles consisting of amphiphilic block copolymer in aqueous medium are receiving considerable attention as gene and drug nanocarriers because of their particular characteristics [1,2,3,4]. Micelles always have a unique core–shell backbone composed of a hydrophilic shell and a hydrophobic core [5]. Hydrophobic drugs can be incorporated into the hydrophobic core of copolymer micelles, whereas the hydrophilic shell can stabilize and protect the drug in the aqueous medium. Furthermore, the hydrophilic shell can prolong the blood circulation time of micelles as a result of steric stabilization, which helps micelles escape mononuclear phagocyte system uptake after intravenous administration [6,7]. Besides, DTX-SA-BSPs copolymer micelles were of low percentage in the injected dose, but could actually accumulate into the tumor, showing the potential of using micelles as a solution for clinical applications [8,9]. Recently, filomicelles are used as highly efficient nanocarriers for drug delivery applications, which provide several advantages, such as deep penetration into tumor, high accumulation into tumor, long circulation time, and enhanced active target delivery [10]. Moreover, the novel filomicelles have high potential as a drug delivery for the nanotherapeutics with improved clinical outcomes [11,12].

In general, amphiphilic block copolymers are obtained by chemical crosslinking of monomers, polymerization of monomers in homogeneous medium, and physical self-assembly of macromolecules [13]. Synthetic polymers are made up of toxic materials, have high immunogenicity, and are nondegradable, limiting their application in drug-delivery systems. Meanwhile, natural polysaccharides have good biocompatibility, biodegradability, and negative immunogenicity. In addition, polysaccharides are easily modifiable and exploitable to improve the targeting capability of carrier systems [14,15]. Docetaxel delivered through pullulan modified with cholesterol and amino groups showed higher lung cancer cell inhibition in vitro and stronger antitumor efficacy in vivo [16]. Hydroxypropyl cellulose was prepared by modifying the cellulose hydroxyl groups with propylene oxide to enhance cellulose solubility and control drug release [17]. Superoxide dismutase-loaded dextran sulfate-cholic acid micelles showed higher stability in acidic medium and sustained drug release up to 100 h in the small intestine, which enhanced the interaction of copolymer micelles with the intestinal membrane and facilitated superoxide dismutase cellular uptake [18]. Stearic acid (SA)-modified chitosan drug-loaded micelles showed significantly higher tumor cell inhibition in vitro compared with free drugs, enhancing drug internalization [19].

Dried root of *Bletilla striata* (Thunb.) *Reichb. f.* is widely used in China to treat skin cracks, swollen tissues, burns, abscesses, and freckles [20]. *B. striata* polysaccharides (BSPs) have many advantages, such as hydrophilicity, biodegradability, nontoxicity, and biocompatibility. BSPs have been extensively used in chemical industries as gels, suspension solutions, and binders as well as in medical and food industries [21,22]. In addition, BSPs have become an excellent candidate for various pharmaceutical applications, such as drug-delivery systems [23]. Li et al. [24] prepared 5-fluorouracil *B. striata* microsphere drug-delivery system with an emulsion-chemical cross-linking method and obtained higher efficacy, lower toxicity, and more long-term effect compared with 5-fluorouracil injection [25]. However, BSPs solubility limits its use as a poorly water-soluble drug carrier. To solve this problem, alkyl, aralkyl, and deoxycholic acid were used to modify water-soluble copolymer to improve its hydrophobic property [26].

**Scheme 1:**
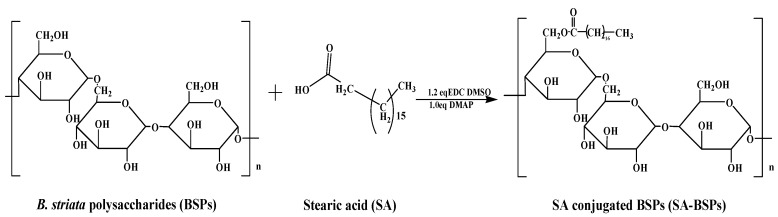
Synthetic route of stearic acid conjugated *B. striata* polysaccharides (SA-BSPs).

**Scheme 2:**
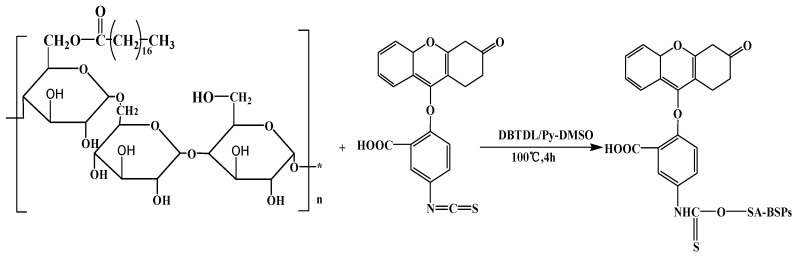
Synthetic route of fluorescein isothiocyanate (FITC) labeled SA-BSPs (FITC-SA-BSPs).

In the present research, BSPs were modified with SA. Scheme 1 presents the synthetic route of SA-conjugated BSPs (SA-BSPs). Scheme 2 presents the synthetic route of fluorescein isothiocyanate (FITC) labeled SA-BSPs (FITC-SA-BSPs). DTX-SA-BSPs copolymer micelles were successfully prepared and characterized in terms of particle size, *zeta* potential, loading capacity (LC), and encapsulation efficiency (EE). Drug release in vitro study was also presented. In vitro anticancer effects of DTX-SA-BSPs copolymer micelles on SW480 human colon cancer cells, HeLa human cervical cancer cells, MCF-7 human breast cancer cells, and HepG2 human liver cancer cells were evaluated. Cellular uptake, apoptosis, toxicity and blood compatibility were also tested.

## 2. Results and Discussion

### 2.1. ^1^H Nuclear Magnetic Resonance (^1^H-NMR) Analysis

**Figure 1:**
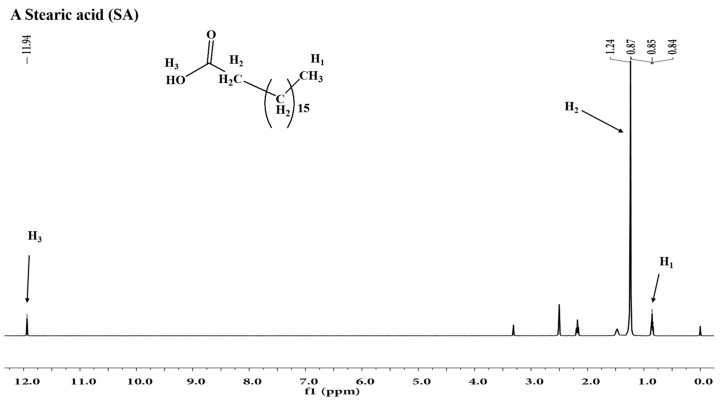

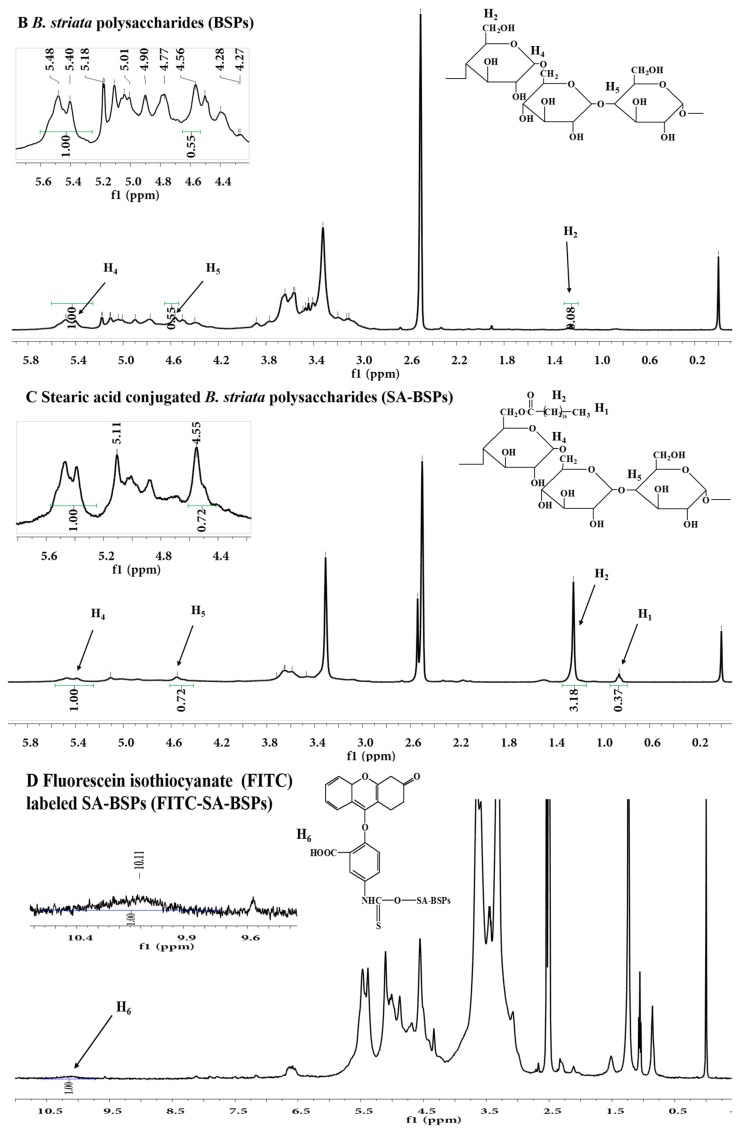
^1^H Nuclear Magnetic Resonance spectra of: SA (**A**); BSPs (**B**); SA-BSPs (**C**); and FITC-SA-BSPs (**D**) in DMSO-*d*_6_*.*

Figure 1 shows the ^1^H-NMR spectra of SA (Figure 1A), BSPs (Figure 1B), SA-BSPs (Figure 1C), and FITC-SA-BSPs (Figure 1D) in DMSO*-d*_6_. δ_1.24_ ppm was the peak of methylene (-CH_2_) protons, and δ_0.85_ ppm was the peak of methyl (-CH_3_) protons. Hydroxyl (-OH) proton signals were observed at δ_4.5–5.6_ ppm in the ^1^H-NMR spectrum of BSPs. δ_5.43_ ppm was the peak of (1 → 6)-linked hydrogen protons, whereas δ_4.55_ ppm was the peak of (1 → 4)-linked hydrogen protons in BSPs. Carboxyl (-COOH) proton signals were observed at δ_10.11_ ppm in the ^1^H-NMR spectrum of FITC-SA-BSPs. The results demonstrated that SA-BSPs and FITC-SA-BSPs copolymers were successfully synthesized, and the results were consistent with previous literature [27,28]. The substituted degree of SA-BSPs was 12.94%, which was calculated from the peak areas (Table 1) of ^1^H-NMR signals. The substituted degree of stearic acid moiety were improved by increasing the mole ratio of SA to BSPs. However, the steric hindrance was enhanced with the increase of the amounts of SA hydrophobic pendant groups conjugated BSPs, which hindered the synthetic reaction and caused the low coupling efficiency. Copolymer micelles hardly filtered through a 0.45 μm membrane with multimodal distribution and turbidity phenomenon when the substituted degree was above 12.94%. Hence, the substituted degree of SA-BSPs_12.94_ was selected for further investigation in our study.

### 2.2. Particle Size and Zeta Potential

Table 2 lists the results of the average diameter and *zeta* potential of copolymer micelles. Compared with that of blank SA-BSPs copolymer micelles, the mean particle diameter increased, whereas *zeta* potential only slightly increased after DTX loading when the drug and SA-BSPs copolymer mass ratio changed from 1:10 to 1:6 (*w*/*w*). The particle size of DTX-SA-BSPs copolymer micelles 125.30 ± 1.89 nm was larger than that of blank SA-BSPs copolymer micelles 96.27 ± 1.21 nm, indicating that particle diameter enlarged with DTX addition because DTX was carried to enter the hydrophobic cores of SA-BSPs copolymer micelles and resulted in the increase in volume of DTX-SA-BSPs copolymer micelles. *Zeta* potential is also an important parameter that reflects either the congregation or electrostatic repulsion of the micelles. Increase of electrostatic repulsive force between micelles can prevent coalescence of the micelles from forming large precipitation, which is useful in maintaining the dispersion stability of these copolymer micelles.

### 2.3. Encapsulation Efficiency and Loading Capacity

Table 2 also presents the effect of different drug and carrier mass ratio on the EE and LC. The results showed that LC increased from 7.87% ± 0.18% to 14.8% ± 0.13% with the increase of drug and carrier mass ratio from 1:10 to 1:6 (*w*/*w*). However, the entrapment efficiency (89.8% ± 0.19%~86.6% ± 0.17%) barely changed with the mass ratio ranging from 1:9 to 1:6. The drug LC and EE of DTX-SA-BSPs copolymer micelles were 14.8% ± 0.13% and 86.6% ± 0.17% when the drug and carrier mass ratio was 1:6.

DTX-SA-BSPs copolymer micelles were successfully fabricated through an emulsion method using docetaxel as the model drug. The results revealed that the maximum LC value of 14.8% ± 0.13% could be reached when drug and carrier ratio was 1:6, whereas its entrapment efficiency slightly decreased. In our previous study, we have successfully prepared DTX-SA-BSPs copolymer micelles, and the values of entrapment efficiency and LC decreased when the drug and carrier mass ration was more than 1:8. The highest percentages of EE and LC were 81.11% ± 0.18% and 9.13% ± 0.17% when the drug and carrier mass ratio was 1:9. DTX-SA-BSPs copolymer micelles with higher entrapment efficiency and LC were prepared with chloroform/ethanol as solvent to dissolve DTX, whereas DTX-SA-BSPs copolymer micelles with lower entrapment efficiency and LC were prepared with ethanol as solvent, which can be attributed to solvent polarity. The solvent polarity of chloroform/ethanol used to solve dissolved DTX in the emulsion method was weaker than that of ethanol alone. Chloroform/ethanol was liable to carry DTX to enter the hydrophobic cores of SA-BSPs copolymer micelles. Therefore, considering the entrapment efficiency and the drug loading capability, we concluded that a drug/carrier ratio of 1:6 be used to prepare DTX-SA-BSPs copolymer micelles, with chloroform/ethanol as solvent to dissolve DTX.

### 2.4. In Vitro Drug Release Study

**Figure 2:**
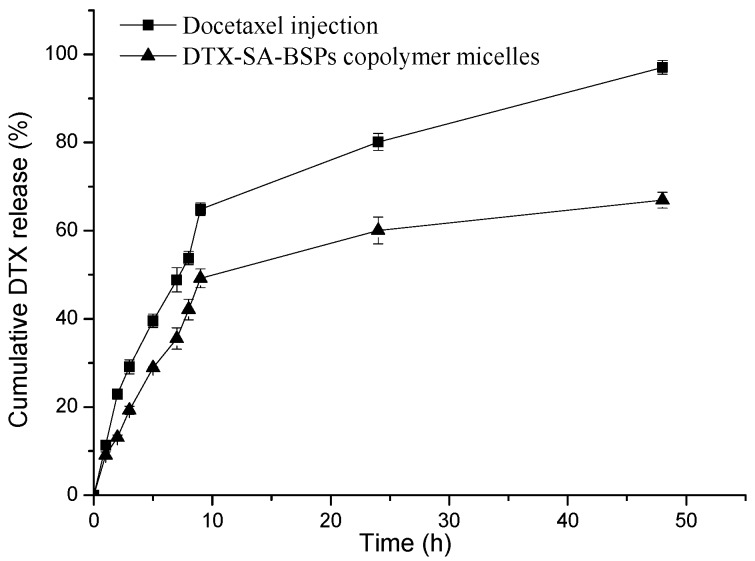
In vitro release profiles of DTX from docetaxel injection (-■-) and DTX-SA-BSPs copolymer micelles (-▲-) in pH 7.4 phosphate-buffered saline containing 0.2% Tween 80 at 37 ± 0.5 °C.

The release profiles of DTX from DTX-SA-BSPs copolymer micelles and docetaxel injection are shown in Figure 2. Docetaxel is a hydrophobic drug, but DTX could be dissolved under sink condition in the phosphate buffer saline (pH 7.4) solution containing 0.2% Tween 80. The amount of DTX released from the micelles increased as a function of time. The release percentage of DTX from docetaxel injection was faster and higher (64.87% ± 1.44%) than that from DTX-SA-BSPs copolymer micelles (49.21% ± 2.15%) in 9 h. The release percentage of DTX from docetaxel injection was approximately 100% after 48 h. The release percentage of DTX from DTX-SA-BSPs copolymer micelles was 60.04% ± 3.06% in the first 24 h and the amount increased to 66.93% ± 1.79% after 48 h. After a rapid release of DTX from DTX-SA-BSPs copolymer micelles during 9 h, a stable plateau was found after 10 h. The results indicated that there might be some free DTX on the surface of the micelles in the beginning of the release process. In the next hours, the DTX release was controlled by the micelles and eventually stopped. The drugs release from nanoparticles depends on these physical and chemical parameters such as drug diffusion rate, partition coefficient between the drug and hydrophobic segment, and copolymer degradation [29]. Based on our experiments, it was likely that the release of DTX on the surface of the micelles directly dissolved in the release medium, while the release of DTX in the micelles was performed through diffusion via micelles.

### 2.5. In Vitro Anticancer Activity

**Figure 3:**
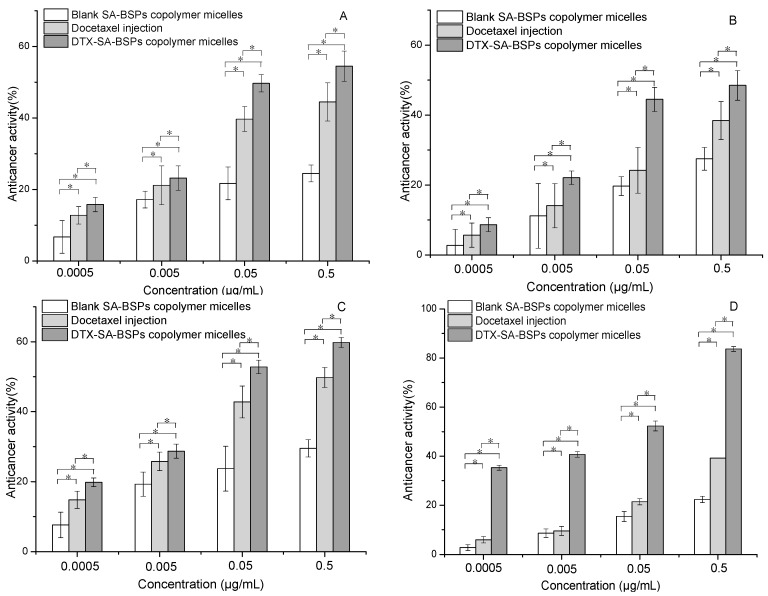
Anticancer activity of SA-BSPs copolymer micelles, docetaxel injection and DTX-SA-BSPs copolymer micelles on: Hela (**A**); SW 480 (**B**); MCF-7 (**C**); and HepG2 (**D**) cells after 72 h incubation (* *p* < 0.05).

HepG2, SW480, MCF-7, and HeLa cells were subjected to MTT assay to evaluate the anticancer activity of blank SA-BSPs copolymer micelles, docetaxel injection, and DTX-SA-BSPs copolymer micelles (Figure 3). As shown in Figure 3, docetaxel injection and DTX-SA-BSPs copolymer micelles apparently had dose-dependent inhibition against HepG2, SW480, MCF-7, and HeLa cells at an equivalent DTX dose from 0.0005 μg/mL to 0.5 μg/mL. SA-BSPs copolymer micelles, docetaxel injection, and DTX-SA-BSPs copolymer micelles presented similar antitumor activity against these four cancer cells. Increased concentration of SA-BSPs copolymer micelles, docetaxel injection, and DTX-SA-BSPs copolymer micelles enhanced their anticancer activity. When SA-BSPs copolymer micelles concentration was 0.5μg/mL, antitumor activity against HepG2 cells decreased. The anticancer activity of blank SA-BSPs copolymer micelles on HeLa, SW480, and MCF-7 cells were 24.5% ± 2.4%, 27.5% ± 3.3%, and 29.5% ± 2.5%, respectively (Figure 3A–C). The blank SA-BSPs copolymer micelles were still biocompatible, although SA-BSPs showed lesser inhibition on cancer cell growth, which may be related to SA-BSPs anticancer activity [30]. When drug concentration was 0.5μg/mL, the antitumor activities of docetaxel injection and DTX-SA-BSPs copolymer micelles on HepG2, HeLa, SW480, and MCF-7cells were 39.2% ± 1.1%, 44.5% ± 5.3%, 38.5% ± 5.4%, and 49.8% ± 2.9%, respectively, whereas the anticancer activity was 83.7% ± 1.0%, 54.5% ± 4.2%, 48.5% ± 4.2%, and 59.8% ± 1.4% at 0.5μg/mL drug concentration, respectively. The anticancer activity of DTX-SA-BSPs copolymer micelles against the four cancer cells was superior to that of docetaxel injection.

Cell viability of DTX-SA-BSPs copolymer micelles had statistically significant difference compared with that of docetaxel injection. The results indicated that DTX-SA-BSPs copolymer micelles were more effective against tumor cells. All data indicated that DTX-SA-BSPs copolymer micelles had better cell inhibition than docetaxel injection. One possible reason was the property of micelles. Drug-loaded micelles have better dispersion, smaller particle size, and larger specific surface area; therefore, the particles have a large surface energy and chemical activity, which are beneficial to enhance the drug-loading effect. At the same time, the micelles are liable to directly penetrate into cancer cells through endocytosis. Copolymer micelles with an approximately 120 nm diameter increased the rate of tumoral uptake by 10–20-fold compared with that of other diameters because of its good retention and permeability [31]. DTX-SA-BSPs copolymer micelles carry DTX into the cancer cells by endocytosis and improve intracellular DTX accumulation [32,33]. Carcinogenic cells possess particular endocytic activity and internalize DTX-SA-BSPs copolymer micelles into the interior of the cell, which improves drug concentration near the action site [34]. In addition, DTX-SA-BSPs copolymer micelles probably avoid the efflux effect of P-glycoprotein (P-gp) pumps. The long and slow DTX release from DTX-SA-BSPs copolymer micelles might be another reason; however, this needs further investigation.

### 2.6. Cellular Uptake

**Figure 4:**
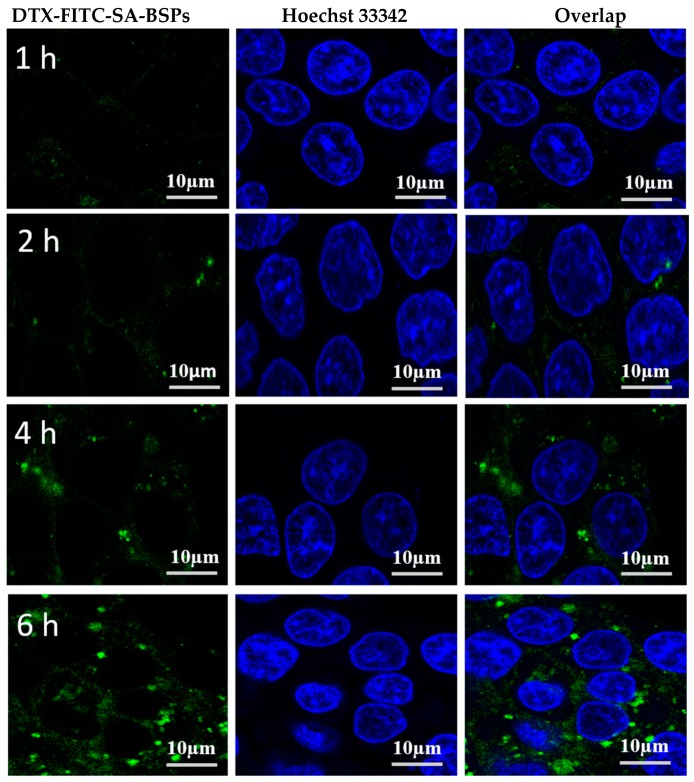
Confocal laser scanning microscopy images of HepG2 cells treated with DTX-FTIC-SA-BSPs copolymer micelles for 1 h, 2 h, 4 h, and 6 h.

Flow cytometry and confocal laser scanning microscopy were used to evaluate the cellular uptake of DTX-FITC-SA-BSPs copolymer micelles. Confocal laser scanning microscopy images were shown in Figure 4. DTX-FITC-SA-BSPs copolymer micelles could be internalized in HepG2 cells and presented green fluorescence in cytoplasm after 1 h incubation. With further incubation for 6 h, cells incubated with DTX-FITC-SA-BSPs copolymer micelles only presented stronger green fluorescence in the cytoplasm. The results indicated the time-dependent cellular uptake pathways of DTX-FITC-SA-BSPs copolymer micelles.

**Figure 5:**
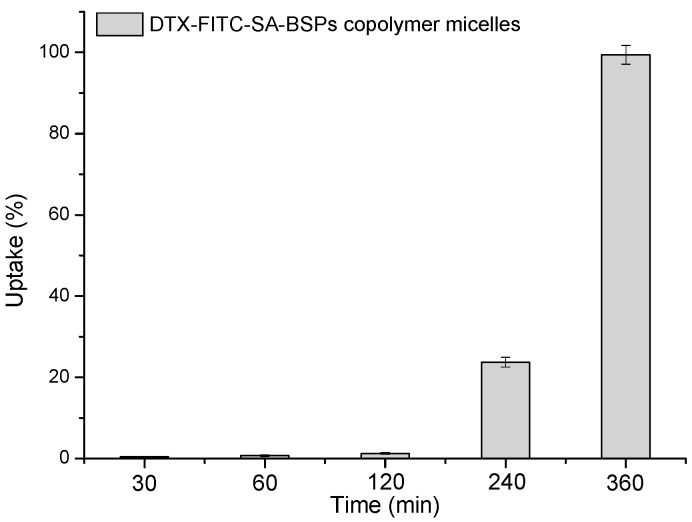
The cellular uptake of DTX-FTIC-SA-BSPs copolymer micelles using flow cytometry assay.

To confirm the quantitative intracellular uptake of DTX-FITC-SA-BSPs copolymer micelles, flow cytometry was used to detect the fluorescence intensity in HepG2 cells, and the cells were treated with DMEM as control. The results are shown in Figure 5. The fluorescence intensity in the HepG2 cell cytoplasm after incubation with DTX-FITC-SA-BSPs copolymer micelles for 30 min, 60 min, 120 min, 240 min, and 360 min were 0.45% ± 0.12%, 0.73% ± 0.19%, 1.31% ± 0.22%, 23.74% ± 1.22%, and 99.41% ± 2.32%, respectively. Fluorescence intensity of FITC in cells increased when the incubation time was prolonged. The fluorescence intensity significantly increased from 1.31% ± 0.22% to 23.74% ± 1.22% when incubating time increased from 120 min to 240 min. The fluorescence intensity of cellular uptake reached 99.41% ± 2.32% when the incubating time was 360 min. The fluorescence intensity was very weak when incubation time was less than 240 min. The results indicated that drug intracellular uptake in micelles improved as incubation time prolonged.

### 2.7. Determining Apoptosis

**Figure 6:**
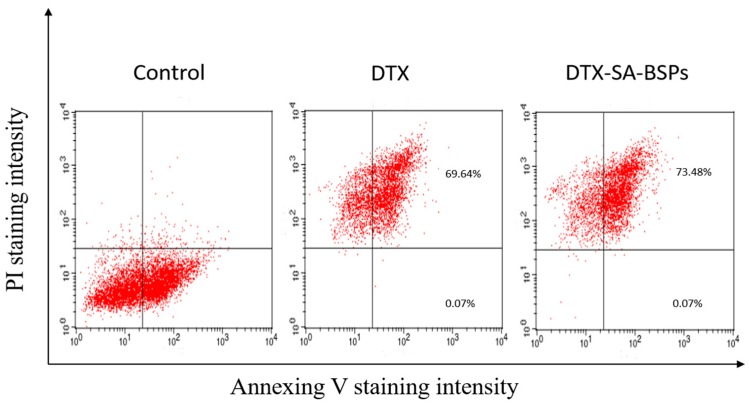
Measurement of cell apoptosis by Annexin V-FITC/PI staining assay. HepG2 cells were treated with 1 mL of 0.5 μg/mL DTX (docetaxel injection) or DTX-SA-BSPs copolymer micelles for 48 h.

Many antitumor drugs function by inducing apoptosis in cancer cells [35]. To evaluate the effect of drug formulations on cell apoptosis, HepG2 cells were incubated with docetaxel injection and DTX-SA-BSPs copolymer micelles 1 mL of 0.5 μg/mL drug concentration for 48 h. We performed an annexin V-FITC/PI double staining assay and determined the apoptotic rate through flow cytometry assay. As shown in Figure 6, annexin-V/PI plots were divided into four areas for distinction: lower right area was early apoptotic cells (Annexin V^+^/PI^−^) and upper right area was late apoptotic cells (Annexin V^+^/PI^+^). The percentage of early apoptotic cells and late apoptotic cells induced by docetaxel injection were 0.07% and 69.64%, whereas the percentage of early apoptotic cells and late apoptotic cells induced by DTX-SA-BSPs copolymer micelles were 0.07% and 73.48%, respectively. These results suggested DTX-SA-BSPs copolymer micelles were more effective for inducing cell apoptosis in HepG2 cells than docetaxel injection. The higher internalization of the drug-loaded copolymer micelles into the cells resulted in better inhibition of anticancer cell growth, induction of cell apoptosis, and cancer cell cycle arrest. Apoptosis results were consistent with their anticancer activity.

### 2.8. In Vitro Hemolysis Assay

**Figure 7:**
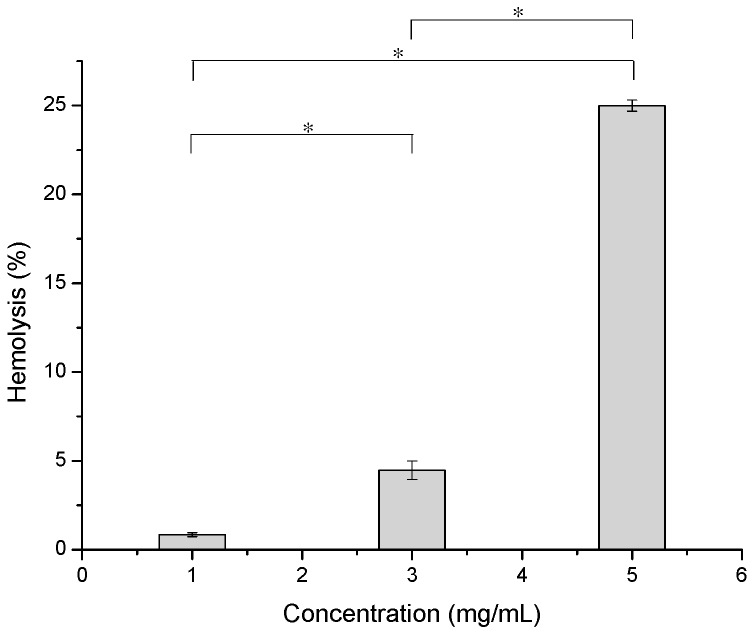
In vitro hemolysis after incubation with SA-BSPs copolymer micelles (* *p* < 0.05).

Hemolysis assay, a major part of biocompatibility test, is the evaluation of the interactions of material with blood to explore possible adverse effects [36]. Hemolysis of rabbit erythrocytes treated with SA-BSPs copolymer is shown in Figure 7. The percent of hemolysis were 0.84% ± 0.20%, 4.47% ± 0.52%, and 24.99% ± 0.32% at 1, 3, and 5 mg/mL micelles concentration, respectively. The percent of hemolysis dramatically increased from 4.47% ± 0.52% to 24.99% ± 0.32% as the micelles concentration increased from 3 mg/mL to 5 mg/mL. However, the percent of hemolysis was below 5% at 1 and 3 mg/mL concentration, which was significantly different from that of 5 mg/mL concentration (*p* < 0.05). Hemolysis induced through high concentration (5 mg/mL) of SA-BSPs copolymer micelles probably resulted from membrane destruction induced by SA-BSPs copolymer micelles [37]. Hemolysis of biomaterials is limited to 5% [38]. Thus, SA-BSPs copolymer micelles showed good blood compatibility below 3 mg/mL concentration, indicating that SA-BSPs copolymer was biocompatible and safe. Therefore, SA-BSPs copolymer micelles may be used as potential biocompatible polymers for cancer chemotherapy.

### 2.9. Toxicity of SA-BSPs Study

**Figure 8:**
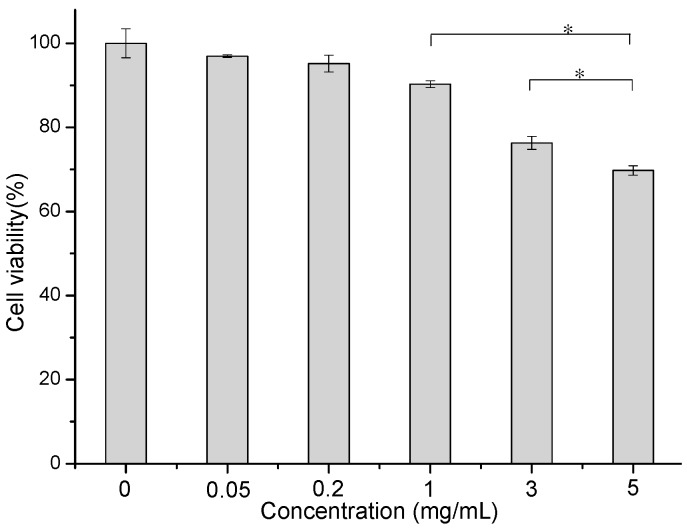
In vitro cytotoxicity after incubation with SA-BSPs copolymer micelles (* *p* < 0.05).

MTT assay was conducted against HUVEC cells to investigate the toxicity of SA-BSPs copolymer micelles. As shown in Figure 8, with the concentrations of SA-BSPs copolymer micelles ranged from 0.05 to 1 mg/mL, the cell viability of HUVEC cells were above 90%, which indicated that the micelles were safe to use as drug carriers below 1 mg/mL. However, the percent of cell viability dramatically decreased from 90.28% ± 0.82% to 76.30% ± 1.53% as the micelles concentration increased from 1 mg/mL to 3 mg/mL. SA-BSPs copolymer micelles presented the similar toxicity trend against HUVEC cells compared with the percent of hemolysis. According to the literature [39], the DTX therapeutic concentration was 6.45 ± 1.18 μg/mL after intravenous infusion 1 h at the dose of 75 mg/m^2^. The corresponding carrier material (SA-BSPs copolymer micelles) concentration was 43.58 ± 7.97 μg/mL. The toxicity and hemolysis results indicated that SA-BSPs copolymer micelles caused no toxicity and hemolysis below 1 mg/ mL. Thus, SA-BSPs copolymer micelles were safe and biocompatible, which was able to be used an encouraging solution for clinical applied [8].

## 3. Experimental Section

### 3.1. Materials and Reagents

Docetaxel injection (Duopafei^®^) was purchased from Qilu Pharmaceutical Co., Ltd. (Jinan, China). Chromatographic-grade acetonitrile and methanol were supplied by Thermo Fisher Scientific (Fair Lawn, NJ, USA). Docetaxel was provided by Shanghai Boyle Chemical Co., Ltd. (Shanghai, China). BSPs were purchased from Shanxi Pioneer Biotech Co., Ltd. (Shanxi, China). 4-Dimethylaminopyridine (DMAP) and 1-ethyl-3-[3-(dimethyl amino) propyl] carbodiimide (EDC) were supplied by Energy Chemical Co., Ltd. (Shanghai, China). SA was purchased from Sinopharm Chemical Reagent Co., Ltd. (Beijing, China). Dulbecco’s modified Eagle medium (DMEM), fetal bovine serum (FBS), trypsin, and phosphate buffer saline (PBS) were all purchased from Thermo Fisher Scientific Co., Ltd. Fluorescein isothiocyanate (FITC), dibutyltin dilaurate, and pyridine were supplied by Aladdin Reagent Co., Ltd. (Shanghai, China). Annexin V-FITC/PI Apoptosis Detection Kit was purchased from Qcbio Science Technologies Ltd. (Shanghai, China). F12K was provided by Wisent Co., Ltd. (Montreal, QC, Canada). All the other reagents were analytical purity grade and commercially obtained.

### 3.2. Synthesis of SA-BSPs and FITC-SA-BSPs Copolymer

SA-BSPs copolymers were synthesized with SA, EDC, and DMAP as shown in Scheme 1. SA (1.8116 g), EDC (1.3801 g), and DMAP (0.7636 g) were added to 15 mL dimethyl sulfoxide (DMSO) solution and stirred for 2 h at 25 °C. BSPs (5.7740 g) dissolved in 20 mL DMSO solution under stirring condition was slowly added dropwise into the mixed solution (15 mL) at 25 °C and continued to react for 48 h at 38 °C. The reaction solution was diluted 10-fold with cold ethanol. The precipitates were recovered through filtration, washed three times with 100 mL ethanol and 100 mL diethyl ether, and dried in a vacuum at 50 °C. SA-BSPs yield was 3.01 g.

A total of 20 mg FITC and 60 mg dibutyltin dilaurate were added to SA-BSPs (200 mg) solution dissolved with DMSO (3 mL) containing 20 μL pyridine. The solution was then heated for 4 h at 100 °C [27]. Other procedures were the same as above. FITC-SA-BSPs copolymer yield was 120 mg.

### 3.3. ^1^H Nuclear Magnetic Resonance (^1^H-NMR) Spectroscopy

^1^H-NMR spectra of the samples (5 mg) dissolved in DMSO-*d*_6_ (500 μL) were determined using a 500 MHz NMR spectrometer (AVIII, Bruker, Rheinstetten, Germany, 500 MHz) at 25 °C [40,41]. All the spectra were performed and processed with Bruker Top Spin version 3.0 software. The substituted degree (DS) of the SA-BSPs group was measured by ^l^H-NMR.

DS was calculated according to the following equation described in [42]:

DS% = (A_δ1.24_/32 + A_δ0.85_/3)/(A_δ5.43_ + A_δ4.55_) × 100% (1)

where A_δ1.24_ was the peak area of methylene protons and A_δ0.85_ was the peak area of methyl protons. A_δ5.43_ was the peak area of hydrogen [H _(1, 6)]_ protons and A_δ4.55_ was the peak area of hydrogen [H _(1, 4)_] protons.

### 3.4. Preparation of DTX-SA-BSPs and DTX-FITC-SA-BSPs Copolymer Micelles

SA-BSPs (50 mg) dissolved in 4 mL DMSO solution was transferred into a cellophane membrane dialysis bag and dialyzed with 0.5 L of deionized water for seven times [41]. Deionized water (0.5 L) was replaced every 2 h for four times and then replaced every 8 h for three times at 100 rpm/min at 25 °C. The copolymer micelle solution filtered through a 0.45 μm membrane filter was adjusted to 50 mL by adding deionized water. Docetaxel (20 mg) was completely dissolved in 10 mL chloroform/absolute ethanol mixture solvent (3:1, *v*/*v*) and slowly added dropwise into copolymer micelle solution under magnetic stirring at 100 rpm/min for 24 h. The solvent was evaporated to harvest the DTX-SA-BSPs copolymer micelles, and the harvested micelles were then adjusted to 100 mL by adding deionized water again.

Creating DTX-FITC-SA-BSPs copolymer micelles were carried out through the same procedure as DTX-SA-BSPs copolymer micelles.

### 3.5. Particle Size and Zeta Potential

The *zeta* potential and particle diameter of the DTX-SA-BSPs copolymer micelles were assessed on dynamic light scattering particle size analyzer with a scattering angle of 90° (Zetasizer Nano ZS, Malvern Instruments, Malvern, UK) at 25 °C [43]. All experiments were carried out in triplicates, and data were expressed as mean values with their standard deviations.

### 3.6. Encapsulation Efficiency and Loading Capacity

The percentage LC of DTX-SA-BSPs copolymer micelles was detected by separating the unentrapped drug from copolymer micelles at 12,000 rpm/min centrifugation speed for 10 min. DTX contents in clear supernatant was analyzed through high-performance liquid chromatography (HPLC, LC-20AT, Shimadzu, Tokyo, Japan). HPLC was equipped with an LC-20AT pump and SPD-20A UV detector controlled through Lab-solution software. EE and LC analysis were performed using a Diamonsil 5 μm C_18_ column (4.6 mm × 250 mm, Dikma, Beijing, China) guarded with a refillable precolumn (C_18_, 4.6 mm × 10 mm, Dikma, Beijing, China) at 30 °C column temperature. The mobile phase was composed of acetonitrile and distilled water at a 60:40 volume ratio. The flow rate was adjusted to 1.0 mL/min, and the wavelength was monitored at 230 nm. All of the experiments were performed in triplicates. The percentage of EE and LC were calculated as follows:

EE (%) = (DTX*_t_* − DTX*_f_*)/DTX*_t_* × 100% (2)

LC (%) = (DTX*_t_* − DTX*_f_*)/weight of copolymer micelles × 100% (3)

where DTX*_t_* was the total weight of docetaxel and DTX*_f_* was the unentrapped docetaxel presented in the supernatant.

### 3.7. In Vitro Drug Release Study

The in vitro release of DTX from DTX-SA-BSPs copolymer micelles was investigated by using the dialysis method. The DTX-SA-BSPs copolymer micelles and docetaxel injection were suspended in 3 mL of distilled water, bringing the final concentration of DTX to 100 μg/mL, and the solution was transferred into a cellophane membrane dialysis bag (8–12 kDa). The dialysis bag was then suspended in 15 mL phosphate buffer saline (PBS, pH 7.4) with 0.2% Tween 80, and subjected to horizontal stirring at a speed of 100 rpm/min at 37 ± 0.5 °C [44]. An aliquot of 5 mL sample was withdrawn at different time points (0, 1, 2, 3, 5, 7, 8, 9, 24 and 48 h) and the solution was compensated with an equal volume of the fresh medium maintained at same temperature. The content of DTX was measured using HPLC. Sink condition was maintained throughout the release periods. All the experiments were performed in triplicate.

### 3.8. In Vitro Anticancer Activity

The anticancer activity of SA-BSPs copolymer micelles, docetaxel injection, and DTX-SA-BSPs copolymer micelles was analyzed through MTT [45] (3-(4,5-dimethylthiazol-2-yl)-2,5-diphenyl tetrazolium bromide) test using HepG2, SW480, HeLa, and MCF-7 cancer cells.

The cells were diluted in certain concentrations and grown in 96-well plates at an initial density of 2 × 10^5^ cells/well with 100 μL DMEM containing 10% FBS and incubated for 24 h at 37 °C in a 5% CO_2_ condition. Cells were treated with SA-BSPs copolymer micelles, docetaxel injection, and DTX-SA-BSPs copolymer micelles at concentrations ranging from 0.0005 μg/mL to 0.5 μg/mL and in pace with five paralleled wells. After 72 h incubation, 20 μL of MTT solution (5 mg/mL) was then added to each well. The medium was removed after incubating for 4 h. A total of 150 μL DMSO was added to dissolve the formazan crystals. The optical density (OD) value was measured at 492 nm using a microplate reader. The percentage of anticancer activity was calculated according to the following equation [46]:

Anticancer activity (%) = (OD_492, control_ − OD_492, sample_)/(OD_492, control_ − OD_492, blank_) × 100 (4)

where OD_492, sample_ was the measurement from the docetaxel injection, blank SA-BSPs copolymer micelles, and DTX-SA-BSPs copolymer micelles; OD_492, control_ was the measurement from the cells treated with incubated solution; and OD_492, blank_ was the incubated solution.

### 3.9. Cellular Uptake In Vitro

HepG2 cells at an initial density of 2 × 10^5^ cells per plate were seeded in 100 μL DMEM containing 10% FBS and incubated for 24 h at 37 °C in a 5% CO_2_ incubator. The cells were incubated for 30 min, 60 min, 120 min, 240 min, and 360 min at 37 °C. The medium was then discarded, and the cells were collected with 0.25% trypsin after washing three times with PBS and centrifuged at 1500 rpm/min for 5 min. Cells were again washed three times with PBS, resuspended in 200 μL PBS, and analyzed through flow cytometry assay [47].

### 3.10. Confocal Laser Scanning Microscopy Observation

Confocal laser scanning microscopy (CLSM) was used to determine the intracellular distribution of DTX-FITC-SA-BSPs copolymer micelles. HepG2 cells were diluted in certain concentrations and grown in 96-well plates at an initial density of 2 × 10^5^ cells/well with 100 μL DMEM containing 10% FBS and incubated for 24 h at 37 °C in a 5% CO_2_ condition. Cells were treated with DTX-FITC-SA-BSPs copolymer micelles for 60 min, 120 min, 240 min, and 360 min at 37 °C and in pace with five paralleled wells. The medium was then discarded, and the cells were washed with PBS three times to remove DTX-FITC-SA-BSPs copolymer micelles which were not ingested by HepG2 cells. Then the nuclei were stained with 15 μL (1 mg/mL) for 15 min. Finally, the cells were rinsed with PBS for three times and incubated with 1 mL DMEM. Fluorescence images of cells were obtained with CLSM (LSM Meta 510 Carl Zeiss, Oberkochen, Germany).

### 3.11. Determining Apoptosis

HepG2 cells were treated with 0.25% trypsin at 37 °C. Digestion was ended by adding DMEM with 10% FBS, and the HepG2 cells were then washed three times with PBS and centrifuged at 1500 rpm/min for 5 min. The cells were harvested and the supernatant was discarded. Annexin V-FITC Apoptosis Detection Kit was used to determine the percentage of apoptosis induced by docetaxel injection or DTX-SA-BSPs copolymer micelles with a volume of 1 mL at 0.5 μg/mL DTX concentration. After 48 h incubation, the supernatant was discarded and the cells were collected, washed three times with PBS, and then resuspended in 500 μL mixture buffer (2 × 10^5^ cells/mL) containing 10 μL propidium iodide and 5 μL annexin V-FITC. The stained cells were detected through flow cytometry assay after incubating for 10–15 min at 25 °C under dark condition [48].

### 3.12. Hemolysis Assay

Briefly, 2 mL PBS was added to 1 mL rabbit blood to separate erythrocytes. Rabbit blood was washed three times with PBS and centrifuged at 3000 rpm/min for 5 min. A total of 200 μL erythrocyte suspension was diluted with 9.8 mL PBS and harvested then 1 mL stock solution of SA-BSPs copolymer micelles (2–10 mg/mL) was added to 1 mL 2% erythrocyte suspension and incubated for 1 h at 37 °C. The unlysed cells were centrifuged at 3000 rpm/min for 5 min, and hemoglobin absorbance in the supernatant was determined at 540 nm through Ultraviolet Visible spectrophotometer. All experiments were done in triplicates, and the percentage of hemolysis was computed as follows:

Hemolysis (%) = (A_test_ − A_negative_)/(A_positive_ − A_negative_) × 100% (5)

where A_test_, A_positive_, and A_negative_ are the absorbance values of the test sample, positive control (water), and negative control (PBS), respectively.

### 3.13. Toxicity of SA-BSPs Study

The cytotoxicity of SA-BSPs copolymer micelles was investigated by MTT assay using HUVEC cells. Cells were seeded onto 96-well plates at an initial density of 5 × 10^4^ cells/well with 100 μL F12K containing 0.4% heparin sodium, 10% FBS, and 1% endothelial cell growth supplement, which were incubated for 24 h at 37 °C in a 5% CO_2_ condition. The medium was placed by samples of various concentrations of SA-BSPs copolymer micelles (0.05, 0.2, 1, 3, and 5 mg/mL) and in pace with five paralleled wells. After 72-h incubation, 20 μL of MTT solution (5 mg/mL) was then added to each well. The medium was removed after incubating for 4 h. A total of 150 μL DMSO was added to dissolve the formazan crystals. The optical density (OD) value was measured at 492 nm using a microplate reader. The cell viability (represented in percent) was calculated according to the following equation:

Cell viability (%) = (OD_492, sample_ − OD_492, blank_)/(OD_492, control_ − OD_492, blank_) × 100 (6)

where OD_492, sample_ was the measurement from the blank SA-BSPs copolymer micelles, OD_492, control_ was the measurement from the cells treated with incubated solution, and OD_492, blank_ was the incubated solution.

## 4. Conclusions

In this study, a novel amphiphilic copolymer was synthesized through covalent attachment of stearic acid to BSPs. The copolymer can easily self-assemble into micelles in aqueous solution. DTX-SA-BSPs copolymer micelles had particle diameter of 125.30 ± 1.9 nm, 86.6% ± 0.17% EE, and 14.8% ± 0.13% LC. The anticancer activity of DTX-SA-BSPs copolymer micelles against four cancer cells, namely, HepG2, HeLa, MCF-7, and SW480, was superior to that of docetaxel injection. The DTX-SA-BSPs copolymer micelles also maintained a constant release rate for a relatively longer time than docetaxel injection. The contents of DTX-FITC-SA-BSPs copolymer micelles in cells had a time-dependent relation. Apoptotic rate of DTX-SA-BSPs copolymer micelles were also higher than that of docetaxel injection. SA-BSPs copolymer had good hemocompatibility and no toxicity at therapeutic concentration, which could be used as an encouraging delivery carrier for poorly water-soluble drugs.

## Figures and Tables

**Table 1:** Peak area of δ_1.24_, δ_0.85_, δ_5.43_ and δ_4.55_^1^H-NMR signals.

Sample	A_δ5.43_	A_δ4.55_	A_δ1.24_	A_δ0.85_
SA-BSPs	0.94	0.64	2.85	0.33

**Table 2:** Characterization of DTX-SA-BSPs copolymer micelles.

Drug/Carrier (*w*/*w*)	EE (%)	LC (%)	Average Diameter (nm)	*Zeta* Potential (mV)
0:10	-	-	96.27 ± 1.21	−35.66 ± 0.28
1:10	78.7 ± 0.12	7.87 ± 0.18	96.54 ± 5.27	−35.46 ± 0.10
1:9	89.8 ± 0.19	9.98 ± 0.16	99.21 ± 3.83	−34.76 ± 0.22
1:7	88.3 ± 0.16	12.5 ± 0.20	121.61 ± 9.81	−28.37 ± 0.12
1:6	86.6 ± 0.17	14.8 ± 0.13	125.30 ± 1.89	−26.92 ± 0.18
